# My School-Boy Days

**Published:** 1864-01

**Authors:** 


### MY SCHOOL-BOY DAYS.

My school-boy days, my school-boy days,
How sweet the light they cast;
How, as on wings of joy, their rays,
Come glimmering o'er the past!
They come as came the joyous gleams
Of sweet but half-forgotten dreams.

My school-boy days, my school-boy days,
They come but once in life;
Like angel-glances on the sea
Of tempest and of strife.
Like some lone minstrel's dying lay,
They echo still, though passed away.

My school-boy days, my school-boy days,
There's magic in the sound;
It calls my young companions up
And sets them smiling round:
With school-boy hopes and school-boy fears,
Its little joys and little tears.

My school-boy days, my school-boy days,
Life looked all sunshine then;
How longed our young ambitious eyes
Impatient to be men!
But have we found in life's dull ways
The joys we lost in school-boy days?

My school-boy days, my school-boy days,
Adieu—in your bright bowers,
Fond memory oft shall while itself
Through life's long, leaden hours,
And echo back, in lonely lays,
The song of school-boy's happy days.

—*Anon.*

